# Strong coupling of multiple plasmon modes and excitons with excitation light controlled active tuning

**DOI:** 10.1515/nanoph-2022-0701

**Published:** 2023-01-26

**Authors:** Yijie Niu, Long Gao, Hongxing Xu, Hong Wei

**Affiliations:** School of Physics and Technology, Center for Nanoscience and Nanotechnology, and Key Laboratory of Artificial Micro- and Nano-structures of Ministry of Education, Wuhan University, Wuhan 430072, China; Beijing National Laboratory for Condensed Matter Physics, Institute of Physics, Chinese Academy of Sciences, Beijing 100190, China; Institute of Microscale Optoelectronics, Shenzhen University, Shenzhen 518060, China; School of Microelectronics, Wuhan University, Wuhan 430072, China; Songshan Lake Materials Laboratory, Dongguan 523808, China

**Keywords:** active tuning, monolayer WSe_2_, photoluminescence, strong coupling, surface plasmon

## Abstract

While the strong coupling between cavity modes and quantum emitters has been investigated in various systems, multiple surface plasmon modes in single nanostructures strongly coupling with excitons are rarely explored. Here, we investigate the strong coupling between three surface plasmon modes in silver nanowires and excitons in monolayer WSe_2_ at room temperature. Four plasmon-exciton polariton (plexciton) states are observed in the scattering spectra. The photoluminescence (PL) spectra of the hybrid system show clear splitting due to strong coupling, and the energies of the emission corresponding to the two lower plexciton states agree with that of the scattering very well. In addition, we show that the plasmon-exciton interaction in this system can be efficiently tuned by controlling the excitation power. These results reveal the fundamental properties of strong coupling between multiple plasmon modes and excitons, deepen the understanding of the correlation between scattering and PL spectra of plasmon-exciton strong coupling systems, and open up a new way to actively control the coupling between plasmonic nanostructures and two-dimensional semiconductors.

## 1 Introduction

When the coherent energy exchange rate between an exciton and an optical cavity is faster than their average dissipation rate, the strong coupling is achieved, which is characterized by the Rabi splitting in frequency domain or Rabi oscillation in time domain. In the past decades, the strong coupling between traditional optical cavities and various quantum emitters has been widely studied [1–5], which shows great potentials for applications in physical and chemical sciences and related technologies. In recent years, the strong coupling of plasmonic nanocavities and excitons has attracted intense interest because surface plasmons (SPs) possess highly confined electromagnetic field with ultrasmall mode volume [6]. This unique property of SPs leads to larger coupling strength and allows for the observation of strong coupling at room temperature at single nanoparticle level [7–10] and even single exciton level [11–17].

Up to now, most works focused on the investigation of strong coupling between only one plasmon mode and one exciton state of emitters, and few studies demonstrated the multimode coupling [18–34]. At the single nanoparticle level, the multimode strong coupling was mainly investigated for one plasmon mode and two exciton states of the same emitters or two different kinds of emitters by scattering spectra [24, 27, 31, 32, 34]. Compared with two modes coupling systems, multimode coupling systems form more polariton states and provide more energy relaxation channels, which is beneficial to further novel applications [35–38].

Monolayers of transition metal dichalcogenides (TMDCs) with atomic thickness, direct band gap transition [39], large exciton binding energy [40], and high optical absorption [41] are ideal candidates for the realization of strong coupling at room temperature. Importantly, monolayer TMDCs at ambient conditions can be easily manipulated by electrostatic gating, thermal control, and optical pumping, exhibiting great capabilities in dynamically tuning plasmon-exciton interactions [42–45]. At the single nanoparticle level, the electrical and thermal control of gold nanrod-WS_2_ coupled system at room temperature [10], and the electrical control of silver nanoprism-WS_2_ coupled system at cryogenic and room temperatures [31] have been demonstrated. Additionally, monolayer TMDCs can also be manipulated by changing the excitation power [46–49], and to our knowledge, such excitation light controlled active tuning for plasmon-exciton strong coupling has not been reported yet.

Here, we firstly investigate the coupling of multiple SP modes on single Ag nanowires (NWs) and excitons in monolayer WSe_2_ by both scattering and PL spectra. The strong coupling of three SP modes and excitons is achieved, resulting in four plexciton branches. A four-coupled-oscillators model is used to analyze the strong coupling behavior. The PL spectra demonstrate the emission mainly from the two lower plexciton states, and the emission peaks agree very well with the energies of the corresponding plexcitons obtained from the scattering spectra. Furthermore, the active control of the strong coupling is achieved by tuning the excitation power, which is demonstrated by PL spectra.

## 2 Results and discussion

**Figure 1:**
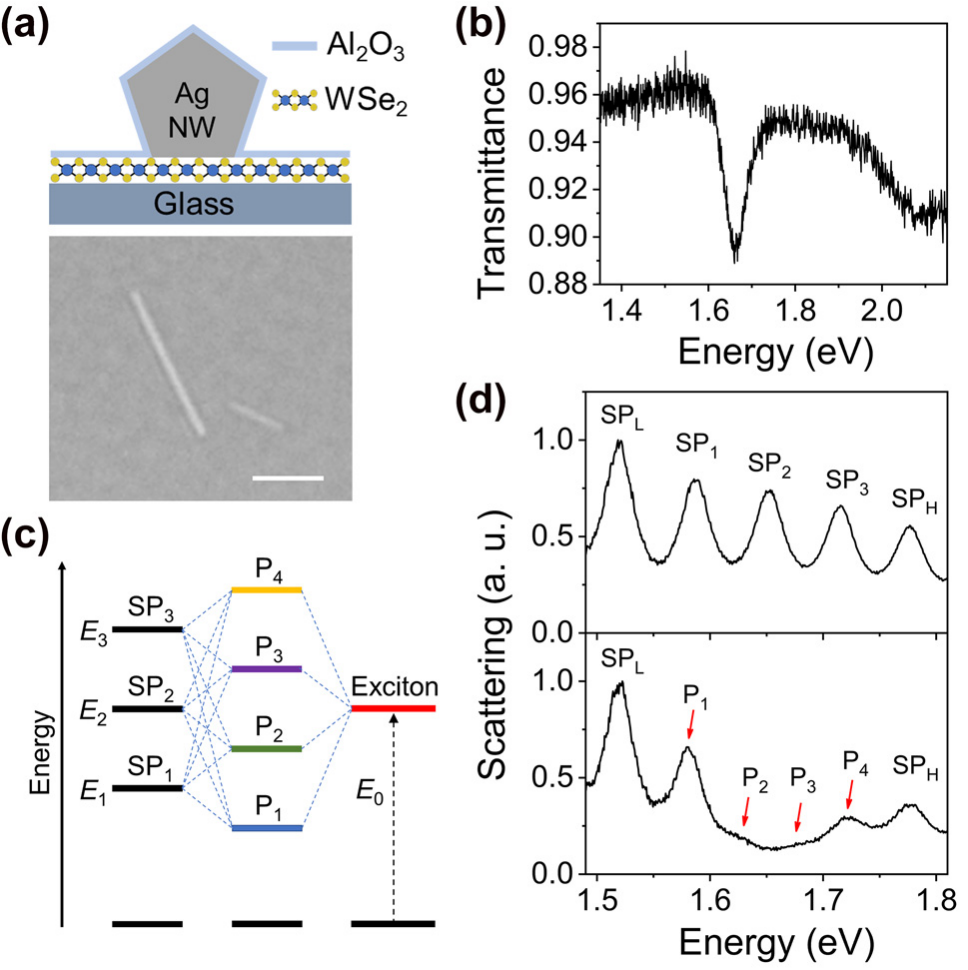
Schematics and characterizations of Ag NW-WSe_2_ coupled system. (a) Schematic of the cross section (top) and optical microscopy image (bottom) of a Ag NW on monolayer WSe_2_. The scale bar is 2 μm. (b) Transmission spectrum of a WSe_2_ monolayer on glass substrate. (c) Schematic representation of the strong coupling between three plasmon modes and one exciton state resulting in four plexciton states. (d) Scattering spectra of Ag NWs without (top) and with (bottom) monolayer WSe_2_. The plexciton peaks are marked with red arrows.

The top panel in Figure 1a shows the schematic of the cross section of the strong coupling system, which consists of a single Ag NW and a WSe_2_ monolayer on a glass substrate. In experiments, after placing the monolayer WSe_2_ and Ag NWs onto the glass substrate, an Al_2_O_3_ layer of initial thickness 5 nm was deposited on top of the sample surface to protect the Ag NWs from degradation (see Methods and Section 1 in Supplementary Material for details). The bottom panel in Figure 1a shows the optical image of one of the Ag NWs with proper dimensions (diameter ∼85 ± 5 nm, length ∼4.39 ± 0.17 μm) on monolayer WSe_2_. The monolayer WSe_2_ on glass substrate shows a transmission dip at *E*_0_ = 1.665 eV (Figure 1b) which corresponds to the A exciton, and the full width at half maximum (FWHM) of the dip is about 55 meV according to a Lorentzian fit. Ag NWs support multiple SP resonance modes that can be easily tuned by modifying the diameter and/or length of the NW, and the refractive index of the surrounding environment. Therefore, Ag NW is an ideal candidate for investigating the strong coupling of multiple SP modes and excitons at the single particle level. When three adjacent SP modes have spectral overlap with excitons, it is possible to construct a three SP modes-excitons coupled system. These three SP modes (named as SP_1_, SP_2_, and SP_3_, with the energy of *E*_1_, *E*_2_, and *E*_3_, respectively) interact with excitons (energy *E*_0_) with coupling strength *g*_1_, *g*_2_, and *g*_3_, respectively, forming four plexciton states P_1_, P_2_, P_3_, and P_4_, as schematically shown in Figure 1c.

The scattering spectra of the coupled systems were measured by focusing the supercontinuum laser light at one end of the NW and collecting the signals at the other end of the NW (see Methods). Due to the small radii of the NWs, only the lowest-order propagating SP mode is excited [50, 51], resulting in the distinct line shapes of Fabry–Pérot resonances in the scattering spectra (see Section 2 in Supplementary Material for the simulation results of the electric field distributions). The top panel in Figure 1d shows the scattering spectrum of a Ag NW on glass substrate. The energy difference between SP_1_, SP_2_, and SP_3_ modes is small enough, so that all these three SP modes can couple with excitons. The SP_L_ and SP_H_ are the SP modes adjacent to SP_1_, SP_2_, and SP_3_ modes, which are far away from the energy of excitons and don’t take part in the interaction. For a Ag NW on monolayer WSe_2_, the spectral features in the middle area becomes largely different, as can be seen from the bottom panel of Figure 1d. The two NWs in Figure 1d have similar lengths and diameters, and the energies of SP_L_ and SP_H_ are similar. Therefore, it is supposed that these two NWs have the same SP resonances, and the SP_2_ mode is resonant with the excitons. In the scattering spectrum of Ag NW-WSe_2_ coupled system, a clear dip is observed at about 1.66 eV, and two peaks appear beside the dip, as marked by P_2_ and P_3_. The energies of P_1_ and P_4_ are redshifted and blueshifted, respectively, compared with that of SP_1_ and SP_3_ modes. These phenomena indicate that all the three SP modes are coupled with the excitons and the four peaks P_1_ to P_4_ correspond to the four plexciton states resulting from the strong coupling of three SP modes and excitons.

**Figure 2:**
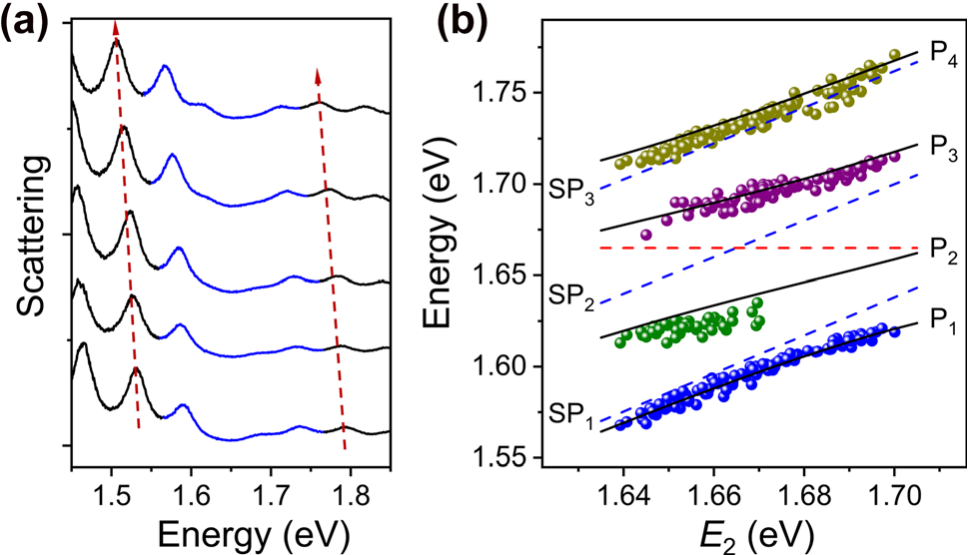
Analyses of scattering spectra for Ag NW-WSe_2_ coupled systems. (a) Scattering spectra of a Ag NW on monolayer WSe_2_ with Al_2_O_3_ of different thickness. The thickness of Al_2_O_3_ is 5 nm, 5.6 nm, 6.6 nm, 8.6 nm, and 10.6 nm from bottom to top. The red arrows mark the redshift of SP_L_ and SP_H_ modes. The strong coupling regions are drawn by blue curves. (b) Energies of P_1_, P_2_, P_3_, and P_4_ (blue, green, purple, and dark yellow dots) as a function of the energy of SP_2_ mode *E*_2_. The black lines are calculated by the Hamiltonian of four-coupled-oscillators model with coupling strength of *g*_1_ = *g*_3_ = 22 meV, and *g*_2_ = 32 meV. The blue dashed lines show the energies of SP_1_ (*E*_1_), SP_2_ (*E*_2_), and SP_3_ (*E*_3_) as a function of *E*_2_. The red dashed line indicates the transmission dip energy of excitons.

Figure 2a shows a set of scattering spectra for a single Ag NW on monolayer WSe_2_ with the Al_2_O_3_ coating thickness increased from bottom to top. The SP modes have an overall redshift with the increase of Al_2_O_3_ thickness, as marked with the red arrows for SP_L_ and SP_H_ modes. We define the energy difference between SP_2_ mode and excitons as detuning. When the SP_2_ mode is close to exciton energy, all the four plexciton branches are clearly observed. When the SP_2_ mode has a large detuning with excitons, the P_2_ (bottom spectrum in Figure 2a, positive detuning) or P_3_ (top spectrum in Figure 2a, negative detuning) are nearly absent in the scattering spectra. This phenomenon is also observed in the strong coupling system of two SP modes and one exciton state [33]. We measured the scattering spectra of dozens of Ag NWs on monolayer WSe_2_ with increasing thickness of Al_2_O_3_ coating layer. The experimental eigenenergies of P_1_, P_2_, P_3_, and P_4_ are obtained by using four Lorentzian peaks to fit the scattering spectra. For the scattering spectra in which the P_2_ or P_3_ is inapparent, three Lorentzian peaks are used for the fitting. The colored dots in Figure 2b show the experimental results of four plexciton branches as a function of the SP_2_ energy *E*_2_.

The energies of SP_1_, SP_2_, and SP_3_ modes are obtained as the average of the values calculated based on their linear relationships with the energies of SP_L_ and SP_H_ modes, since SP_L_ and SP_H_ modes do not couple with excitons (see Section 3 in Supplementary Material). The calculated results of *E*_1_, *E*_2_, and *E*_3_ as a function of *E*_2_ are plotted with the blue dashed lines in Figure 2b. Using a four-coupled-oscillators model (see Methods) and adjusting the coupling strength *g*_1_, *g*_2_, and *g*_3_, the eigenenergies of the plexciton branches are obtained. As shown by the black lines in Figure 2b, the calculated dispersions agree well with the experimental results.

When the SP_2_ mode is in resonance with excitons, the minimal splitting between the two plexciton branches P_2_ and P_3_ is Ω ≈ 56 meV. In accordance with the criterion for the strong coupling of two oscillators, the minimal splitting between adjacent plexciton branches larger than their mean linewidth can be used as the criterion for the multimode strong coupling. The linewidths of P_2_ and P_3_ obtained from the four-coupled-oscillators model are $\gamma_{P_{2}}=37.9$ meV and $\gamma_{P_{3}}=37.8$ meV, respectively (see Section 4 in Supplementary Material). It is clear that $\Omega >\left(\gamma_{P_{2}}+\gamma_{P_{3}}\right)/2$ , indicating the strong coupling regime is reached. The calculated contribution fractions of SP_1_, SP_2_, SP_3_, and excitons for four plexciton branches are shown in Figure S4. As can be seen from Figure S4b and c, when the SP_2_ mode is largely detuned with respect to the exciton energy, the excitons make a large contribution to P_2_ (for positive detuning) or P_3_ (for negative detuning). The increased weight of excitons for P_2_ and P_3_ leads to their smaller scattering cross sections and thus lower intensities in the scattering spectra.

**Figure 3:**
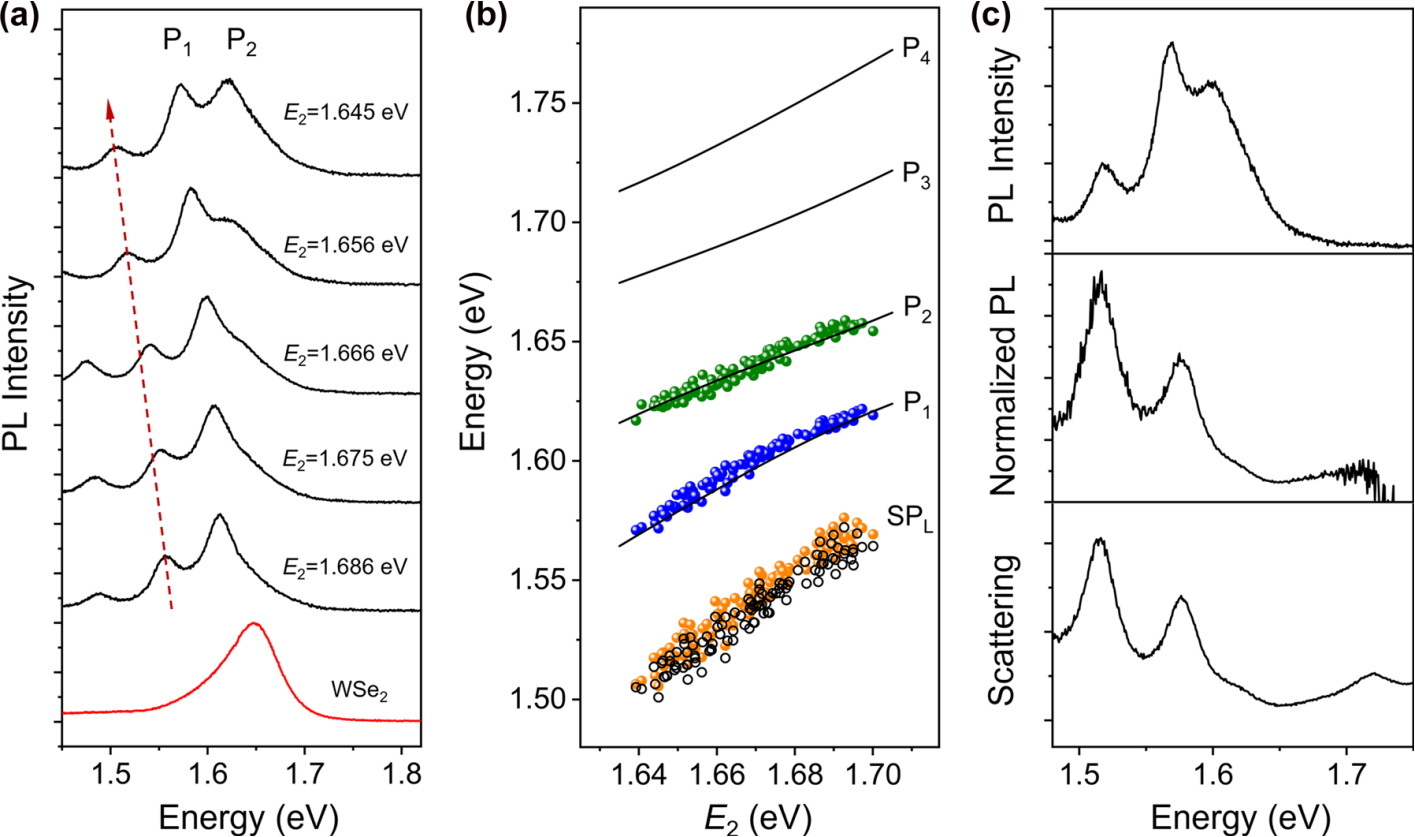
Analyses of PL spectra for Ag NW-WSe_2_ coupled systems. (a) PL spectra of Ag NWs on monolayer WSe_2_ with different *E*_2_. The red arrow marks the redshift of SP_L_ mode. The two plexciton peaks are labeled as P_1_ and P_2_. The PL spectrum of bare monolayer WSe_2_ without Ag NW is plotted at the bottom. (b) Energies of fitting peaks in the experimental PL spectra as a function of *E*_2_ (orange, blue, and green dots). The black lines are the calculated results for the scattering in Figure 2b. The black hollow dots are the energies of SP_L_ mode extracted from the corresponding scattering spectra. (c) Top: PL spectrum for a Ag NW-WSe_2_ coupled system when SP_2_ mode is close to exciton energy (*E*_2_ = 1.650 eV). Middle: normalized PL spectrum resulting from dividing the top spectrum by the PL spectrum of bare WSe_2_. Bottom: scattering spectrum for the same coupled system.

The PL spectra of the coupled systems were also measured by setting the excitation and detection positions at the two opposite ends of the NWs. The polarization of 532 nm laser light is perpendicular to the NWs and the excitation power is about 0.076 mW. Figure 3a shows the PL spectra when the SP_2_ mode scans across the exciton energy from positive detuning to negative detuning (see Figure S6 in Supplementary Material for the corresponding scattering spectra). As can be seen, the PL spectra of the coupled systems are largely different from that of bare WSe_2_ (bottom spectrum in Figure 3a), and multiple peaks are observed. The three peaks in the top spectrum in Figure 3a correspond to SP_L_, P_1_, and P_2_, respectively (see Section 6 in Supplementary Material). As *E*_2_ is decreased, the SP_L_ peak is redshifted, as marked by the red arrow. Meanwhile, the redshift of P_1_ is also clearly observed. At large positive detuning (large *E*_2_ value), the intensity of P_2_ is lower compared with P_1_. With the decrease of *E*_2_, the ratio of P_2_ to P_1_ intensities is increased, and the intensities of these two plexciton peaks become comparable.

The PL spectra are fitted by multiple Lorentzian peaks (see Figure S9 in Supplementary Material), and the energies of the three higher-energy fitting peaks are plotted as a function of the SP_2_ energy *E*_2_ (orange, blue, and green dots in Figure 3b). As can be seen, the blue and green dots agree well with the black lines corresponding to P_1_ and P_2_ for scattering spectra, respectively. The energies of orange dots agree with the energies of SP_L_ mode in the corresponding scattering spectra (black hollow dots in Figure 3b). When the PL spectrum of the coupled system is divided by that of bare WSe_2_, the resulting normalized PL spectrum shows the same profile as the corresponding scattering spectrum from the same coupled system, as shown in Figure 3c. The similarity of the two spectra demonstrates that the four plexciton states resulting from the strong coupling of three SP modes and excitons are also manifested in the PL spectrum of the coupled system [33]. These results clearly demonstrate that the spectra of PL emitted through the scattering of SP-exciton hybrid modes are closely related to the scattering spectra of the strong coupling system, and both the PL and scattering spectra can reflect the plexciton states formed due to the strong coupling.

**Figure 4:**
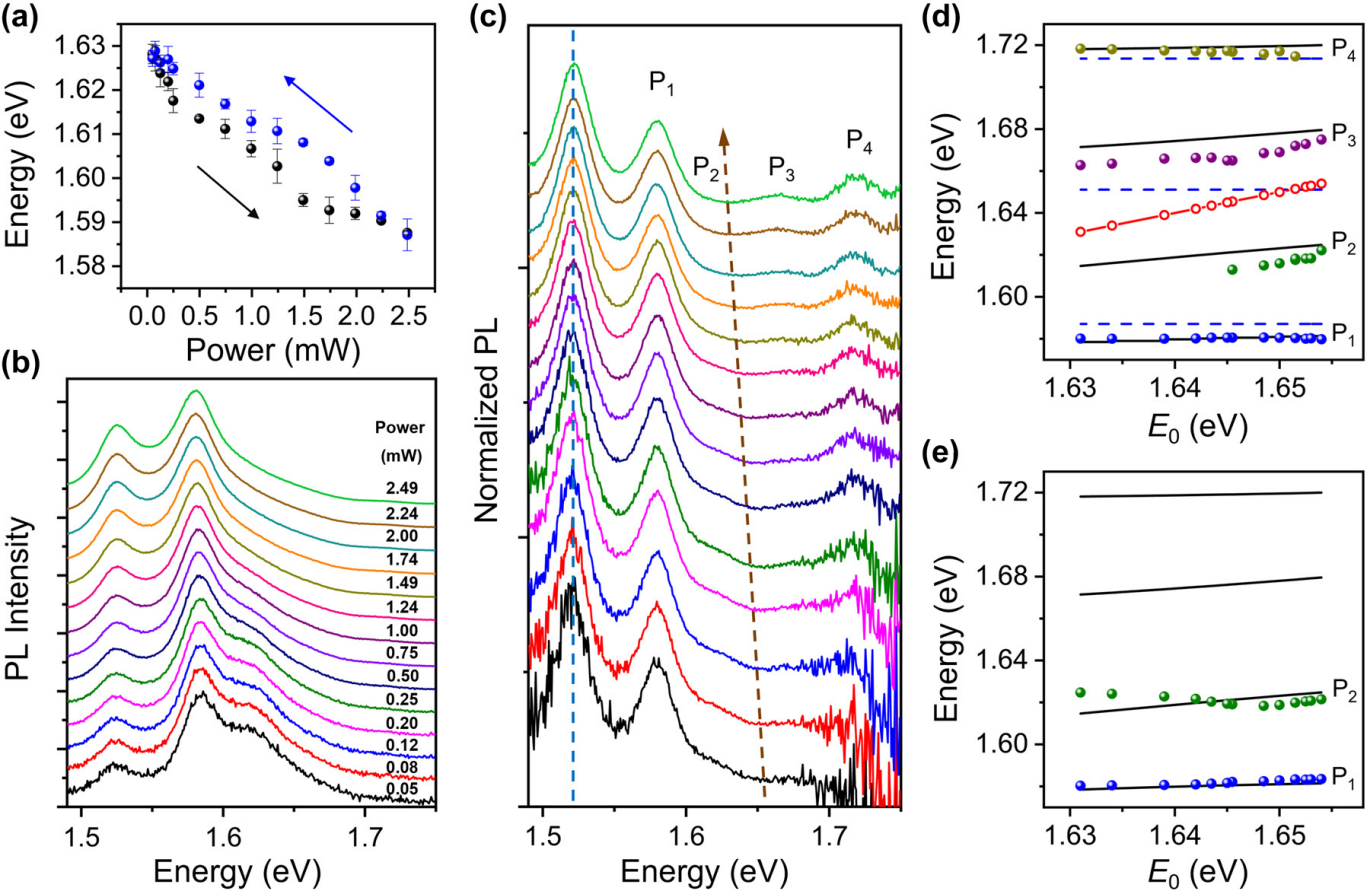
Active control over strong coupling by tuning excitation power. (a) PL peak energy of bare monolayer WSe_2_ as a function of excitation power. The black and blue arrows show the processes of increasing and decreasing the excitation power, respectively. The black and blue dots are the average values obtained from the spectra measured at three positions on the same WSe_2_ monolayer, and the error bars represent the standard deviation. (b) PL spectra of a Ag NW on monolayer WSe_2_ with excitation power increased from bottom to top. The thickness of Al_2_O_3_ is 15 nm. (c) Normalized PL spectra obtained by dividing the spectra in (b) by the PL spectra of bare WSe_2_ at the same excitation power. The redshift of the dip is marked with the brown arrow, and the SP_L_ mode is marked with the blue dashed line. The four plexciton peaks are labeled as P_1_, P_2_, P_3_, and P_4_. (d) Energies of P_1_, P_2_, P_3_, and P_4_ (blue, green, purple, and dark yellow dots) extracted from (c) as a function of the dip energy *E*_0_. The black lines are calculated by the Hamiltonian with coupling strength of *g*_1_ = *g*_3_ = 18 meV, and *g*_2_ = 30 meV. The blue dashed lines show the energies of SP_1_, SP_2_, and SP_3_. The red solid line with hollow dots shows the energy of excitons extracted from (c). (e) Energies of P_1_ and P_2_ (blue and green dots) extracted from (b) as a function of *E*_0_. The black lines are the same as in (d).

At last, we demonstrate the active control by excitation light over the strong coupling. It is found that the peak energy of the PL spectra of bare monolayer WSe_2_ can be reversibly modulated by increasing or decreasing the excitation power of 532 nm laser (see Figures 4a and S10 in Supplementary Material). The redshift of the PL peak with increasing excitation power can be attributed to the lift of Fermi level and the creation of trions resulting from the increased density of photoionized carriers [46–49]. Additionally, as the PL peak is redshifted with the increase of temperature [10, 43, 47], the laser-induced thermal effect may also contribute to the redshift of the PL spectra. To check whether the thermal effect can influence the SP resonance energies, we measured the scattering spectra of a Ag NW on glass substrate excited by the supercontinuum laser light under the illumination of 532 nm laser light polarized parallel to the NW. The spectra show that the SP resonance energies are almost independent on the power of the 532 nm laser light (see Figure S11 in Supplementary Material), which indicates that the contribution of thermal effect to the PL spectral redshift of monolayer WSe_2_ may be small. In the following measurements for the coupled system, the polarization of the 532 nm laser light is set to be perpendicular to the NW, which can further suppress the influence of thermal effect induced by the SPs on the Ag NW [52].

Figure 4b demonstrates a set of PL spectra from a coupled system when the excitation power is increased from 0.05 mW to 2.49 mW. The profiles of these spectra are similar to that in Figure 3a. Therefore, it can be determined that the three peaks in the spectra correspond to SP_L_, P_1_, and P_2_, respectively. With the increase of the excitation power, the intensity of P_2_ peak becomes relatively lower compared to that of P_1_ peak. Since the normalized PL spectra show the same profiles as the corresponding scattering spectra, the active control can be analyzed by the normalized PL spectra obtained by dividing the PL spectra of the coupled system by the PL spectra of the bare WSe_2_ at the same excitation power. The energy of the dip in the normalized PL spectra (marked by a brown arrow in Figure 4c) can be regarded as the transmission dip energy of excitons for each excitation power. With the increase of the excitation power, the dip energy is decreased (see Figure S12 in Supplementary Material). The energy of SP_L_ mode keeps constant when increasing the excitation power, as marked with the blue dashed line in Figure 4c, which further confirms that the SP resonance energies are not affected by the excitation light. Using the energies of SP_L_ and SP_H_ obtained from the scattering spectrum and the linear relationships in Figure S3, the energies of SP_1_, SP_2_, and SP_3_ modes are determined to be *E*_1_ = 1.587 eV, *E*_2_ = 1.651 eV, and *E*_3_ = 1.714 eV. With the increase of the excitation power, the energy of excitons scans across the SP_2_ mode from the less negative detuning to the positive detuning, which leads to the decrease of the intensity of P_2_ relative to P_1_ in Figure 4b and c and the increase of the intensity of P_3_ relative to P_2_ in Figure 4c. These results are consistent with Figures 2 and 3 which show the intensities of P_2_ and P_3_ are low at positive and negative detuning, respectively. By using Lorentzian peaks to fit the normalized PL spectra in Figure 4c and the PL spectra in Figure 4b, the experimental energies of the plexcitons are obtained, which are plotted as a function of the dip energy *E*_0_ (colored solid dots in Figure 4d and e). The black lines in Figure 4d and e show the eigenenergies calculated by the four-coupled-oscillators model. All these results demonstrate the active control over the strong coupling.

## 3 Conclusions

We have investigated the strong coupling of three SP modes and excitons by both scattering and PL spectra in the coupled systems of Ag NWs and monolayer WSe_2_. Four plexciton branches resulting from the strong coupling are obtained from the scattering spectra. In the PL spectra, peak splitting due to strong coupling is clearly observed. The dispersions of the two lower plexciton branches obtained from the PL spectra agree very well with that from the scattering spectra. Furthermore, we have demonstrated excitation light controlled active tuning in this multimode strong coupling system. These results not only confirm the connection between PL and scattering spectra of plasmon-exciton strong coupling systems, but also provide more possibilities to manipulate the strong coupling. The coupling system of plasmonic NWs and two-dimensional TMDCs offers a versatile platform for exploring plasmon-exciton interactions and related applications.

## 4 Methods

### 4.1 Sample preparation

The WSe_2_ monolayers on 300 nm SiO_2_/Si substrate (1 cm × 1 cm) were purchased from a company (Nanjing Muke Nanotechnology). They were transferred to a glass substrate in the following way. Firstly, the solution of polystyrene (PS) in toluene (50 mg/mL) was spin-coated onto the SiO_2_/Si substrate with monolayer WSe_2_ at a speed of 3000 rpm for 60 s. Secondly, the substrate was placed on a hot plate (80 °C) for 15 min to ensure that the WSe_2_ monolayers were tightly attached to the PS layer. Thirdly, the sample was immersed in NaOH solution (2 mol/L, 60 °C) for 2 min to etch the interface between the silica substrate and the monolayer WSe_2_. Subsequently, the sample was slowly immersed into deionized water, where the PS layer with the attached WSe_2_ would be separated from the silica substrate. The PS piece was fished up onto a clean glass substrate, and was placed on a hot plate (130 °C) for 1 h to ensure that the WSe_2_ monolayers were tightly attached to the glass. Then the PS layer was removed by toluene (room temperature, 2 h). Finally, the sample was washed by ethanol (30 min) and deionized water (5 min), and dried by high purity nitrogen blow.

After transferring the monolayer WSe_2_ onto the glass substrate, chemically synthesized Ag NWs were drop-casted onto the monolayer WSe_2_. Then an Al_2_O_3_ layer of 5 nm thickness was deposited onto the sample by using atomic layer deposition method. Additional Al_2_O_3_ was deposited after the optical measurements to tune the SP resonance energies. For the experiments of laser power tuned PL, a sample with an Al_2_O_3_ layer of 15 nm thickness was used, because we found that the sample with thicker Al_2_O_3_ coating is more stable under high power laser illumination. Figure S1a shows the scanning electron microscopy image of a Ag NW with proper dimensions. The monolayer nature of the WSe_2_ is confirmed by measuring the Raman spectra (Figure S1b). The deposition of additional Al_2_O_3_ doesn’t induce noticeable change to the PL spectra of monolayer WSe_2_ (Figure S1c).

### 4.2 Optical measurements

To measure the scattering spectra of the coupled systems, supercontinuum laser light (repetition rate 10 MHz) was focused onto one end of the NW from the glass side through an oil immersion objective (100×, NA 1.49), and the emitted light from the other end of the NW was collected by the same objective and directed to a spectrometer (Princeton Instruments, Acton SP2500). For PL measurements, the configuration for excitation and detection is the same as that for the scattering spectra. The coupled systems were excited by 532 nm continuous-wave laser light with polarization perpendicular to the NW, and a 633 nm long wave pass filter was inserted in the detection path to block the laser light.

### 4.3 Coupled oscillators model

The strong coupling of three SP modes and excitons can be described by a four-coupled-oscillators model:

$$\begin{array}{ll} & \left(\begin{array}{cccc} E_{1}-i\gamma_{1}/2 & 0 & 0 & g_{1}\\ 0 & E_{2}-i\gamma_{2}/2 & 0 & g_{2}\\ 0 & 0 & E_{3}-i\gamma_{3}/2 & g_{3}\\ g_{1} & g_{2} & g_{3} & E_{0}-i\gamma_{0}/2 \end{array}\right)\\ & \;\times \left(\begin{array}{c} \alpha \\ \beta \\ \delta \\ \varphi \end{array}\right)=E\left(\begin{array}{c} \alpha \\ \beta \\ \delta \\ \varphi \end{array}\right) \end{array}$$

where *E*_1_, *E*_2_, *E*_3_, and *E*_0_ are the energies of the SP_1_ mode, SP_2_ mode, SP_3_ mode, and excitons, respectively; *γ*_1_, *γ*_2_, *γ*_3_, and *γ*_0_ are the FWHMs corresponding to *E*_1_, *E*_2_, *E*_3_, and *E*_0_, respectively; *g*_1_, *g*_2_, and *g*_3_ are the coupling strengths of SP_1_ mode with excitons, SP_2_ mode with excitons, and SP_3_ mode with excitons, respectively; *E* is the eigenvalue of the energy for corresponding plexcitons; *α*, *β*, *δ*, and *φ* are the Hopfield coefficients which satisfy ${\left|\alpha \right|}^{2}+{\left|\beta \right|}^{2}+{\left|\delta \right|}^{2}+{\left|\varphi \right|}^{2}=1$ .

## Supplementary Material

Supplementary Material Details
