# Erratum to: Strong coupling of multiple plasmon modes and excitons with excitation light controlled active tuning

**DOI:** 10.1515/nanoph-2023-0263

**Published:** 2023-05-17

**Authors:** Yijie Niu, Long Gao, Hongxing Xu, Hong Wei

**Affiliations:** School of Physics and Technology, Center for Nanoscience and Nanotechnology, and Key Laboratory of Artificial Micro- and Nano-structures of Ministry of Education, Wuhan University, Wuhan 430072, China; Beijing National Laboratory for Condensed Matter Physics, Institute of Physics, Chinese Academy of Sciences, Beijing 100190, China; Institute of Microscale Optoelectronics, Shenzhen University, Shenzhen 518060, China; School of Microelectronics, Wuhan University, Wuhan 430072, China; Songshan Lake Materials Laboratory, Dongguan 523808, China

After the publication of this article [1], the authors found that the energy range of the photoluminescence (PL) spectrum in the top panel of Figure 3c is incorrect. The correct version of Figure 3 is shown below, and the caption of the figure remains the same. This error does not affect the conclusions of the article.

**Figure 3:**
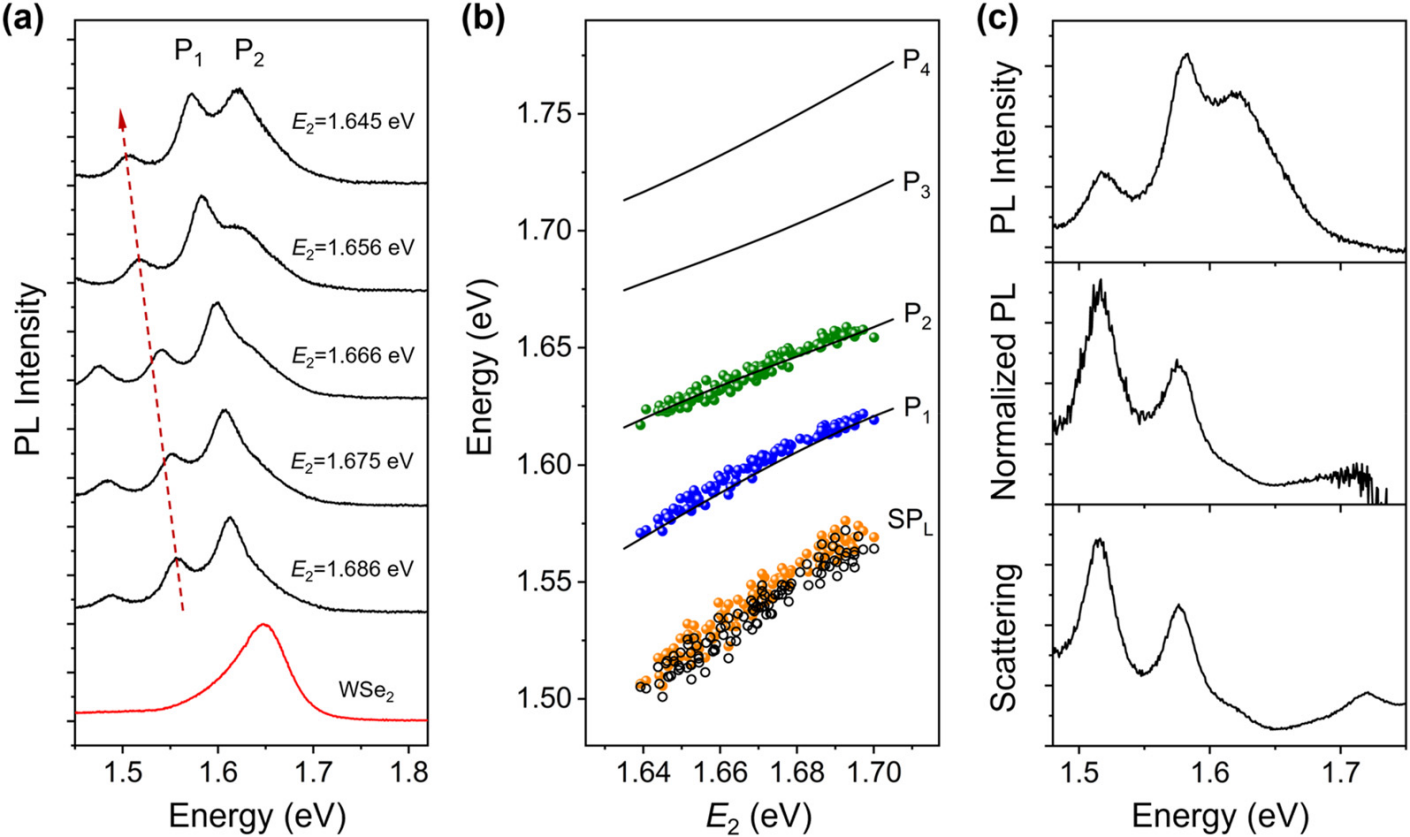
Analyses of PL spectra for Ag NW-WSe_2_ coupled systems. (a) PL spectra of Ag NWs on monolayer WSe_2_ with different *E*_2_. The red arrow marks the redshift of SP_L_ mode. The two plexciton peaks are labeled as P_1_ and P_2_. The PL spectrum of bare monolayer WSe_2_ without Ag NW is plotted at the bottom. (b) Energies of fitting peaks in the experimental PL spectra as a function of *E*_2_ (orange, blue, and green dots). The black lines are the calculated results for the scattering in Figure 2b. The black hollow dots are the energies of SP_L_ mode extracted from the corresponding scattering spectra. (c) Top: PL spectrum for a Ag NW-WSe_2_ coupled system when SP_2_ mode is close to exciton energy (*E*_2_ = 1.650 eV). Middle: Normalized PL spectrum resulting from dividing the top spectrum by the PL spectrum of bare WSe_2_. Bottom: Scattering spectrum for the same coupled system.
