# Supplementary material for: Strong coupling of multiple plasmon modes and excitons with excitation light controlled active tuning
Source: Nanophotonics. 2023 Jan 26;12(4):735–42. doi: 10.1515/nanoph-2022-0701 (PMC11636511; doi:10.1515/nanoph-2022-0701)
Supplement: Supplementary file 1 — Supplementary Material Details [file j_nanoph-2022-0701_suppl_001.docx]

Supplementary Material

Strong Coupling of Multiple Plasmon Modes and Excitons with Excitation Light Controlled Active Tuning

Yijie Niu,^1,2^ Long Gao,^2,3^ Hongxing Xu,^1,4,^* and Hong Wei^2,5,^*

^1^School of Physics and Technology, Center for Nanoscience and Nanotechnology, and Key Laboratory of Artificial Micro- and Nano-structures of Ministry of Education, Wuhan University, Wuhan 430072, China

^2^Beijing National Laboratory for Condensed Matter Physics, Institute of Physics, Chinese Academy of Sciences, Beijing 100190, China

^3^Institute of Microscale Optoelectronics, Shenzhen University, Shenzhen 518060, China

^4^School of Microelectronics, Wuhan University, Wuhan 430072, China

^5^Songshan Lake Materials Laboratory, Dongguan 523808, China

*E-mail: hxxu@whu.edu.cn; weihong@iphy.ac.cn

1. Characterizations of Ag NWs and monolayer WSe_2_

The Ag NWs were synthesized by using a polyol method [1]. Figure S1a shows the scanning electron microscopy (SEM) image of a Ag NW with proper dimensions on Si substrate. The Raman spectra of the WSe_2_ layer were measured by using a Raman spectrometer system (inVia, Renishaw) with an excitation laser of 514 nm. As shown in Figure S1b, there is no peak around 308 cm^-1^, indicating the monolayer nature of the WSe_2_ [2]. Figure S1c shows the PL spectra of monolayer WSe_2_ with Al_2_O_3_ coating of different thickness, which demonstrates that the deposition of additional Al_2_O_3_ doesn’t induce noticeable change to the PL spectra of monolayer WSe_2_.

**
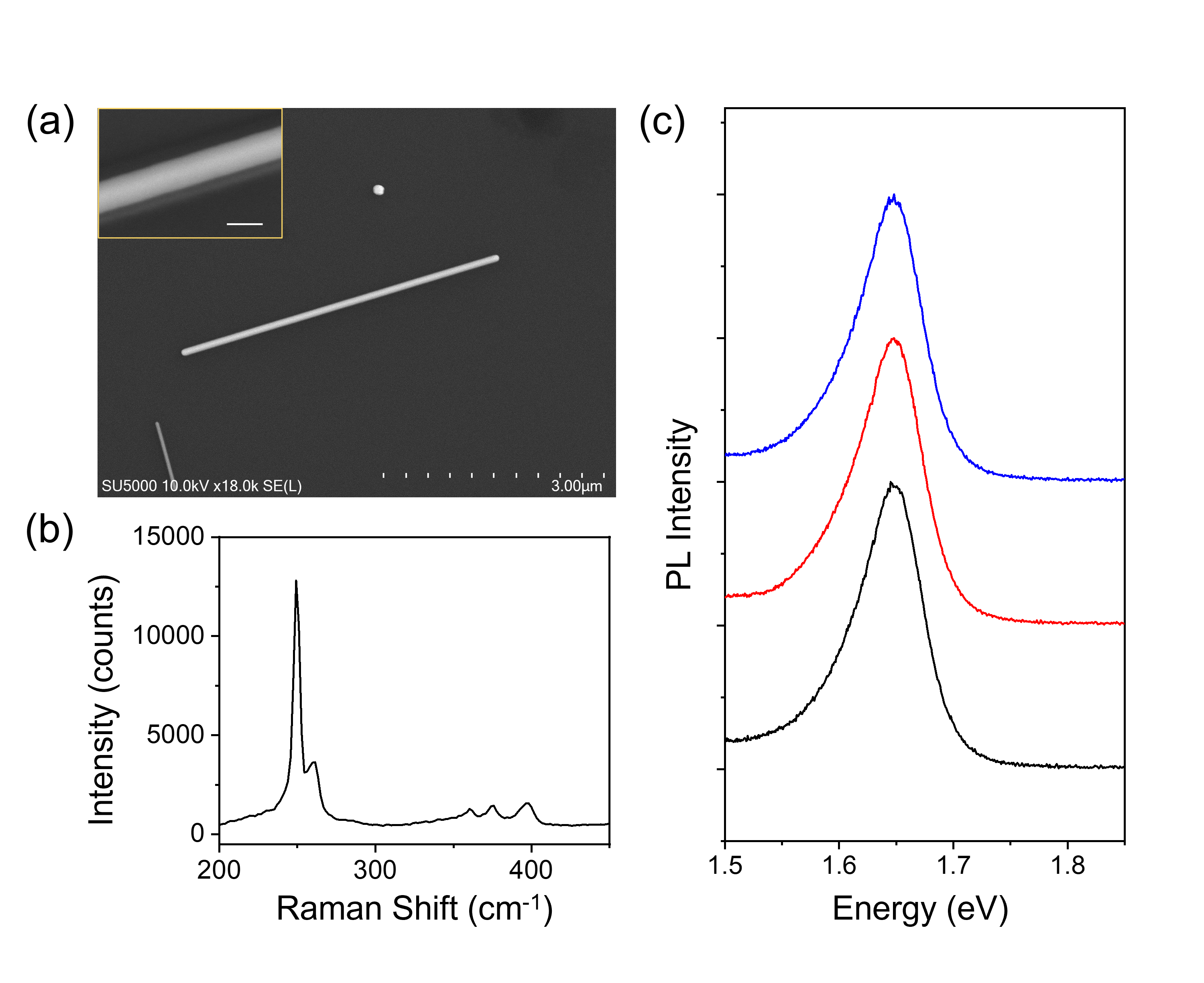
**

**Figure S1.** (a) SEM image of a typical Ag NW on Si substrate (diameter 88 nm, length 4.51 μm). Inset: SEM image with higher magnification of the same Ag NW. The scale bar is 100 nm. (b) Raman spectrum of monolayer WSe_2_. (c) PL spectra of monolayer WSe_2_ with Al_2_O_3_ coating thickness of 5 nm, 8.6 nm, and 10.6 nm from bottom to top.

1. Simulation results of electric field distributions

Figure S2a shows the simulation result of the total electric field distribution of a Ag NW coated with Al_2_O_3_ on glass substrate excited by a Gaussian beam incident on the left end of the NW from the glass side. The light is polarized along the long axis of the NW. The Ag NW can serve as a Fabry–Pérot resonator and the periodic field distribution of the standing wave formed by the propagating SPs is observed. Figure S2b shows the distributions of the total electric field and in-plane electric field component of the lowest order propagating SP mode obtained by mode analysis for a Ag NW with diameter of 85 nm. All simulations were performed by the finite element method (COMSOL Multiphysics).


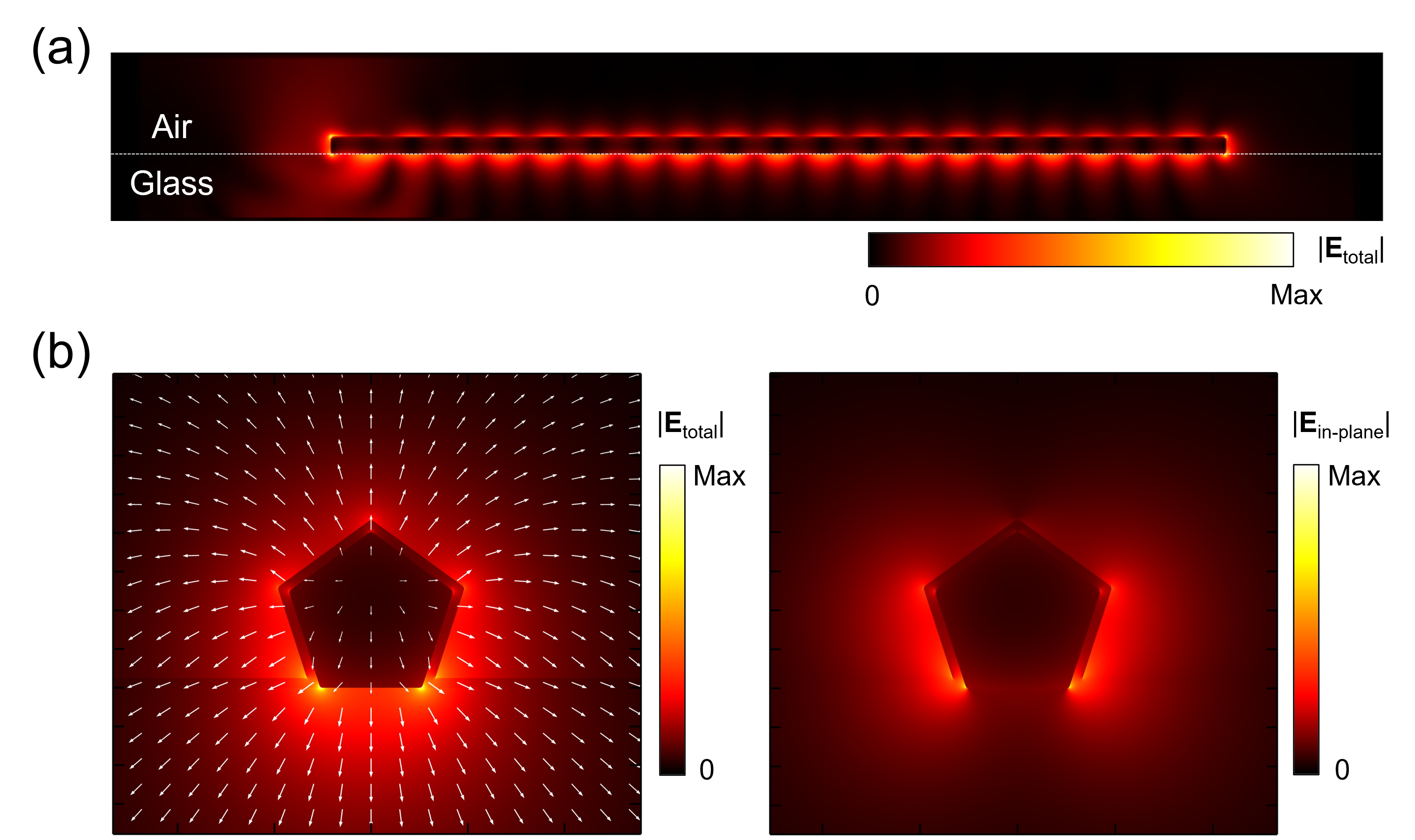


**Figure S2.** (a) Total electric field distribution on the vertical central plane of a Ag NW on glass substrate excited by a Gaussian beam on the left end of the NW. The length and diameter of the NW are 4.25 μm and 85 nm, respectively. (b) Total (left) and in-plane (right) electric field distributions of the lowest order mode supported by a Ag NW on glass substrate. The electric field lines are marked with arrows. The two color bars in (b) show the same amplitude range. The NW diameter is 85 nm. The Al_2_O_3_ thickness and wavelength are 5 nm and 745 nm, respectively, for both (a) and (b).

1. Energies of SP modes extracted from scattering spectra

We experimentally measured the scattering spectra of uncoupled Ag NWs which have similar length and same number of SP modes as those Ag NWs coupled with monolayer WSe_2_. The energies of SP_1_, SP_2_, SP_3_, and SP_H_ (SP_L_) are extracted from the scattering spectra according to Lorentzian fits and plotted as a function of SP_L_ (SP_H_), as shown in Figure S3a (Figure S3b). As can be seen, the energies of these SP modes are approximately in linear relationships. For the Ag NW-WSe_2_ coupled systems, the energies of SP_L_ and SP_H_ are extracted from the scattering spectra, and the energies of SP_1_, SP_2_, and SP_3_ are calculated by the linear relationships in Figure S3a and b. The two sets of energies of SP_1_, SP_2_, and SP_3_ obtained from SP_L_ and SP_H_ are averaged. Figure S3c shows the average energies of SP_1_, SP_2_, and SP_3_ as a function of the energy of SP_2_.


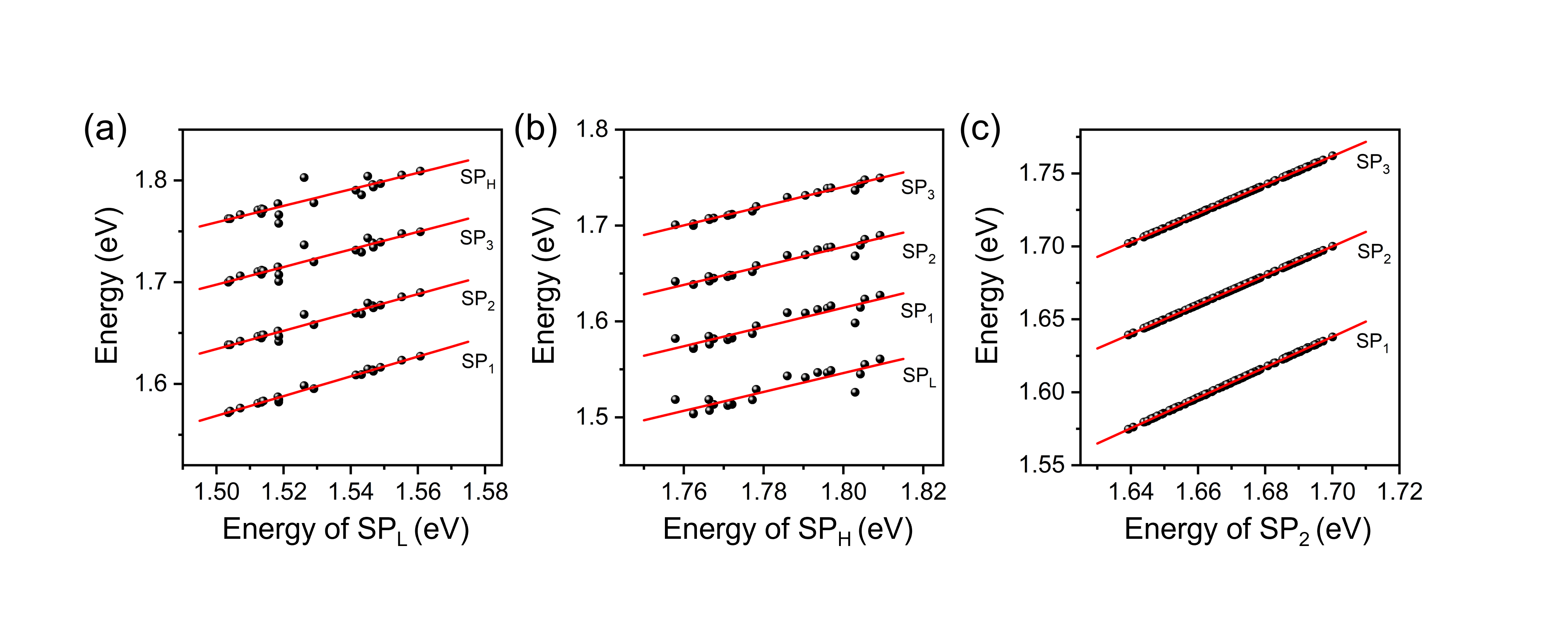


**Figure S3.** (a) Energies of SP_1_, SP_2_, SP_3_, and SP_H_ as a function of the energy of SP_L_. (b) Energies of SP_L_, SP_1_, SP_2_, and SP_3_ as a function of the energy of SP_H_. (c) Energies of SP_1_, SP_2_, and SP_3_ of the Ag NW-WSe_2_ coupled systems as a function of the energy of SP_2_. The red lines are liner fits to the black dots.

1. Calculated fractions of SP modes and excitons and linewidths of four plexciton branches from four-coupled-oscillators model


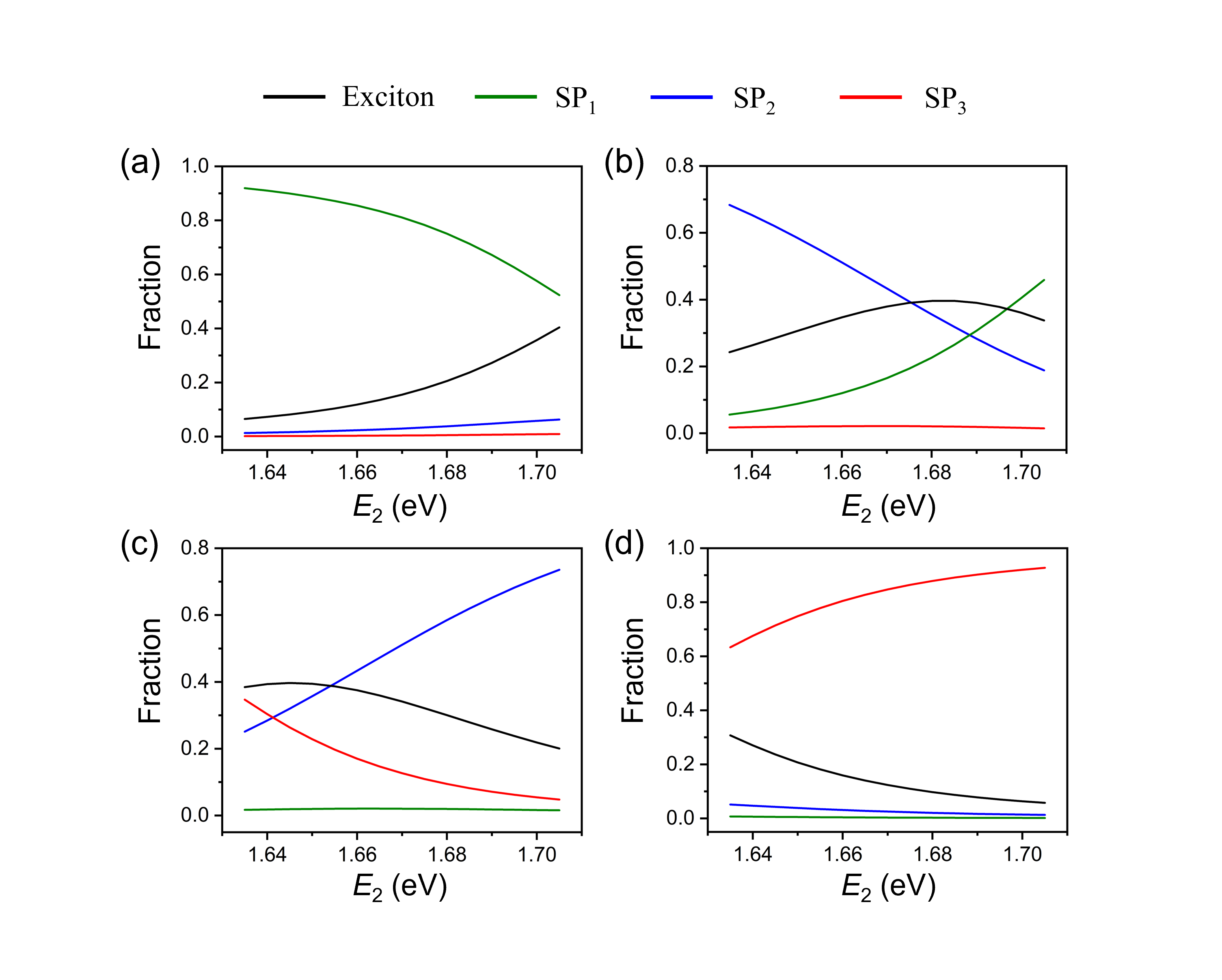


**Figure S4.** Calculated results of the mixing fractions of SP_1_ mode, SP_2_ mode, SP_3_ mode, and excitons for P_1_ (a), P_2_ (b), P_3_ (c), and P_4_ (d) as a function of *E*_2_.

Figure S4 shows the calculated contribution fractions of SP_1_ mode, SP_2_ mode, SP_3_ mode, and excitons for four plexciton branches (P_1_, P_2_, P_3_, and P_4_) as a function of the energy of SP_2_ mode *E*_2_. As shown in Figure S4a (Figure S4d), P_1_ (P_4_) is mainly contributed by SP_1_ (SP_3_) and excitons, and the weights of SP_2_ and SP_3_ (SP_1_) are small and increased when *E*_2_ is increased (decreased). For P_2_ (P_3_), as shown in Figure S4b (Figure S4c), SP_1_ (SP_3_), SP_2_, and excitons all contribute significantly to the strong coupling, and the maximum contribution of excitons is reached at *E*_2_ larger (smaller) than the exciton energy. The weight of SP_3_ (SP_1_) for P_2_ (P_3_) is small and gets to the maximum when *E*_2_ is close to the exciton energy.


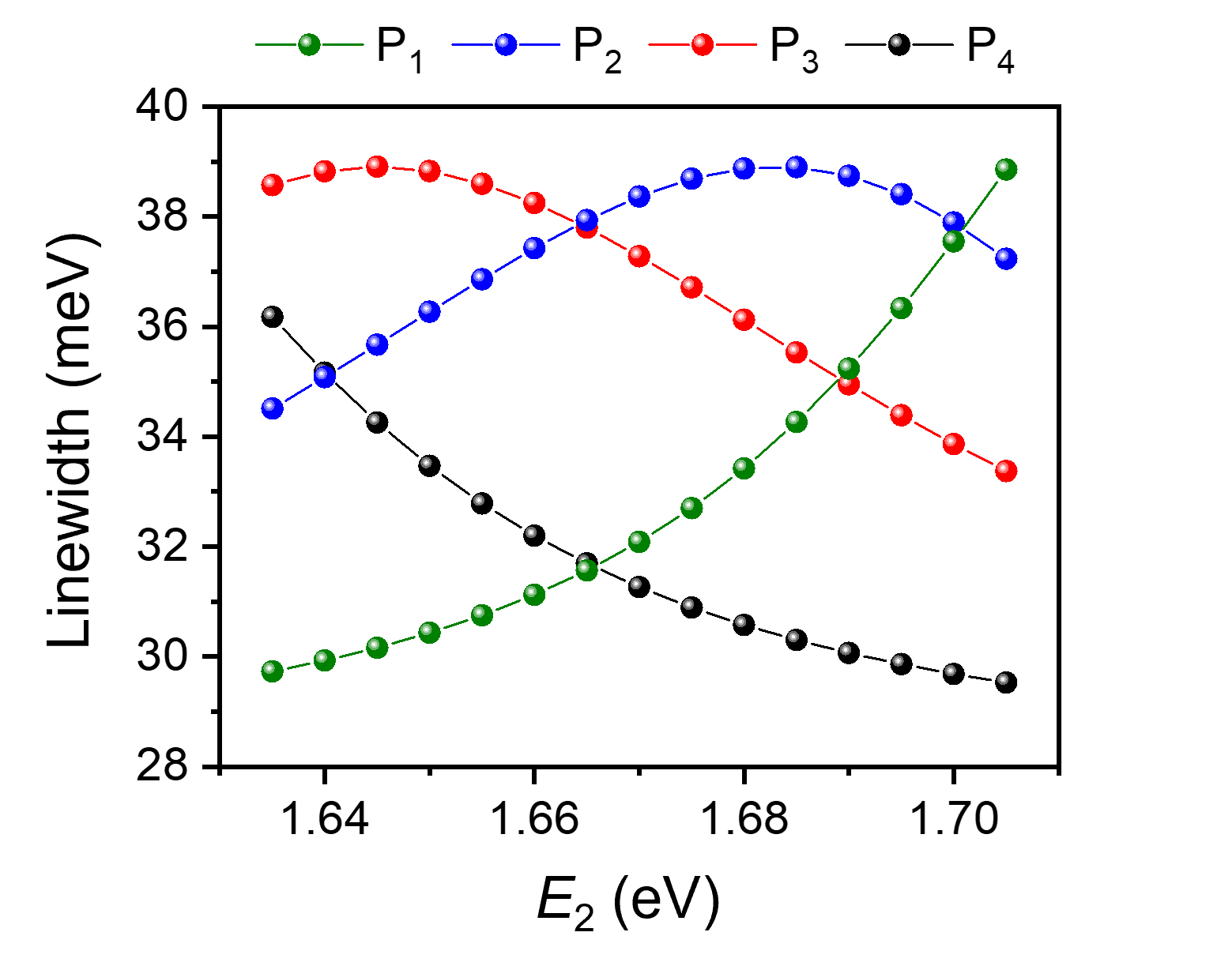


**Figure S5.** Calculated results of the linewidths of four plexciton branches.

Figure S5 shows the linewidths of four plexciton branches as a function of *E*_2_ calculated by the four-coupled-oscillators model. The linewidth of each plexciton branch consists of the contributions of SP_1_ mode, SP_2_ mode, SP_3_ mode, and excitons, and can be written as $\gamma_{i}=F_{i,\mathrm{SP}_{1}}\gamma_{1} + F_{i,\mathrm{SP}_{2}}\gamma_{2}+F_{i,\mathrm{SP}_{3}}\gamma_{3}+F_{i,X}\gamma_{0}$, where the subscript *i* represents P_1_, P_2_, P_3_, and P_4_; $F_{i,\mathrm{SP}_{1}}$, $F_{i,\mathrm{SP}_{2}}$, $F_{i,\mathrm{SP}_{3}}$, and $F_{i,X}$ are the fractions of SP_1_ mode, SP_2_ mode, SP_3_ mode, and excitons, respectively, for each plexciton branch, which are shown in Figure S4; $\gamma_{1}$, $\gamma_{2}$, $\gamma_{3}$, and $\gamma_{0}$ are the linewidths of SP_1_ mode, SP_2_ mode, SP_3_ mode, and excitons, respectively. For the Ag NWs on glass substrate, $\gamma_{1}\approx\gamma_{2}\approx\gamma_{3}\approx28$ meV. The linewidth of excitons is $\gamma_{0}\approx55$ meV.

In the two-coupled-oscillators model for the strong coupling of one SP mode and one exciton state, the linewidths of both upper and lower plexciton states at zero detuning are equal to the mean linewidth of the SP mode and the exciton state. The criterion for the strong coupling of one SP mode and one exciton state is that the Rabi splitting at zero detuning is larger than their mean linewidth, i.e., larger than the mean linewidth of two plexciton states. Accordingly, the criterion for the strong coupling of multiple modes can be written as the minimal splitting between adjacent plexciton branches larger than their mean linewidth. When the SP_2_ mode is in resonance with excitons ($E_{2}=1.665$ eV), the linewidths of P_2_ and P_3_ are $\gamma_{P_{2}}=37.9$ meV and $\gamma_{P_{3}}=37.8$ meV, respectively. The splitting between P_2_ and P_3_ (56 meV) is larger than $(\gamma_{P_{2}}+\gamma_{P_{3}})/2$, confirming that the strong coupling regime is reached.

1. Scattering spectra corresponding to Figure 3a


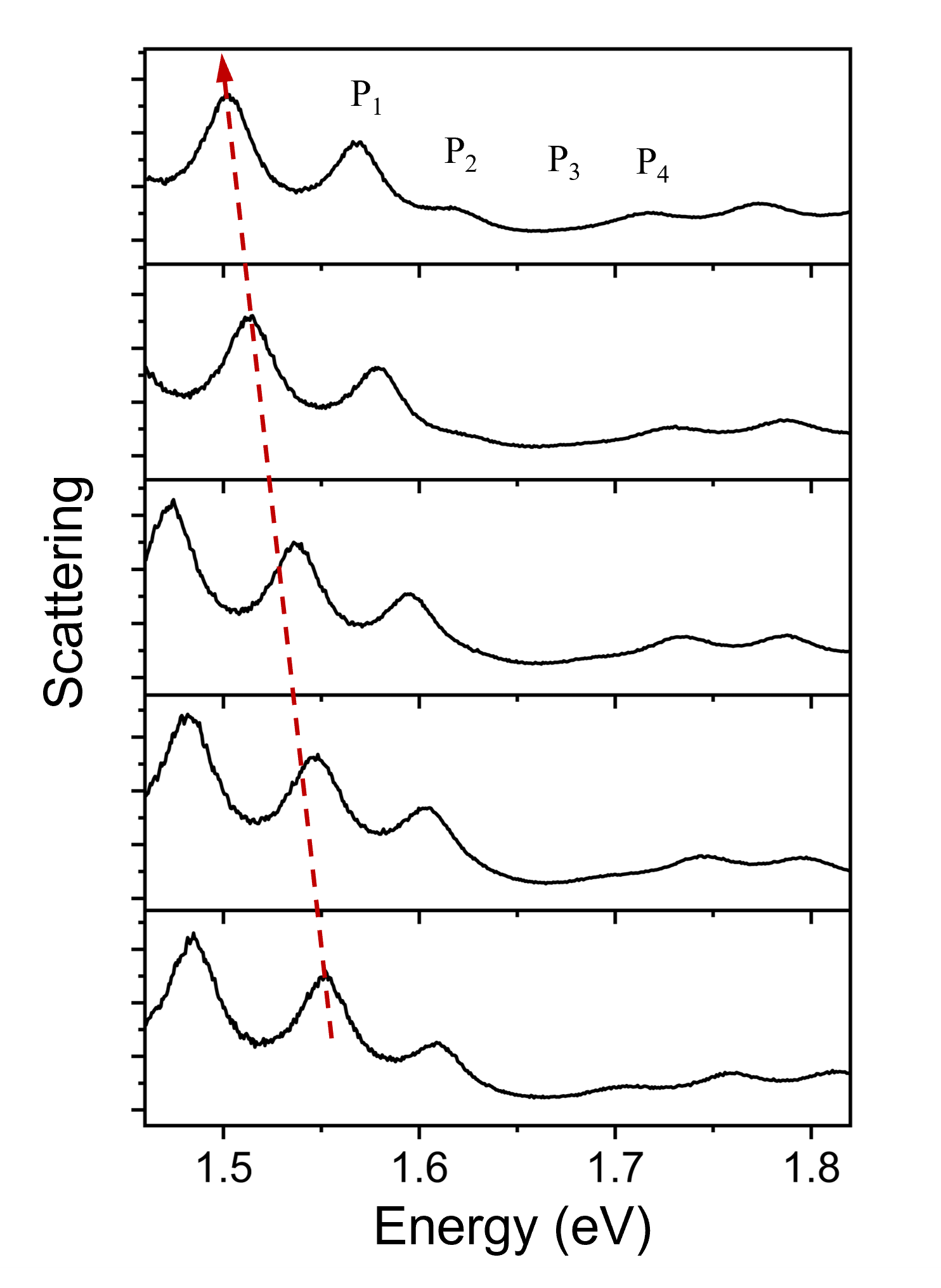


**Figure S6.** Scattering spectra of Ag NW-WSe_2_ coupled systems corresponding to Figure 3a. The red arrows mark the redshift of SP_L_ mode. The four plexciton peaks are labelled as P_1_, P_2_, P_3_, and P_4_.

1. Analyses of PL spectra


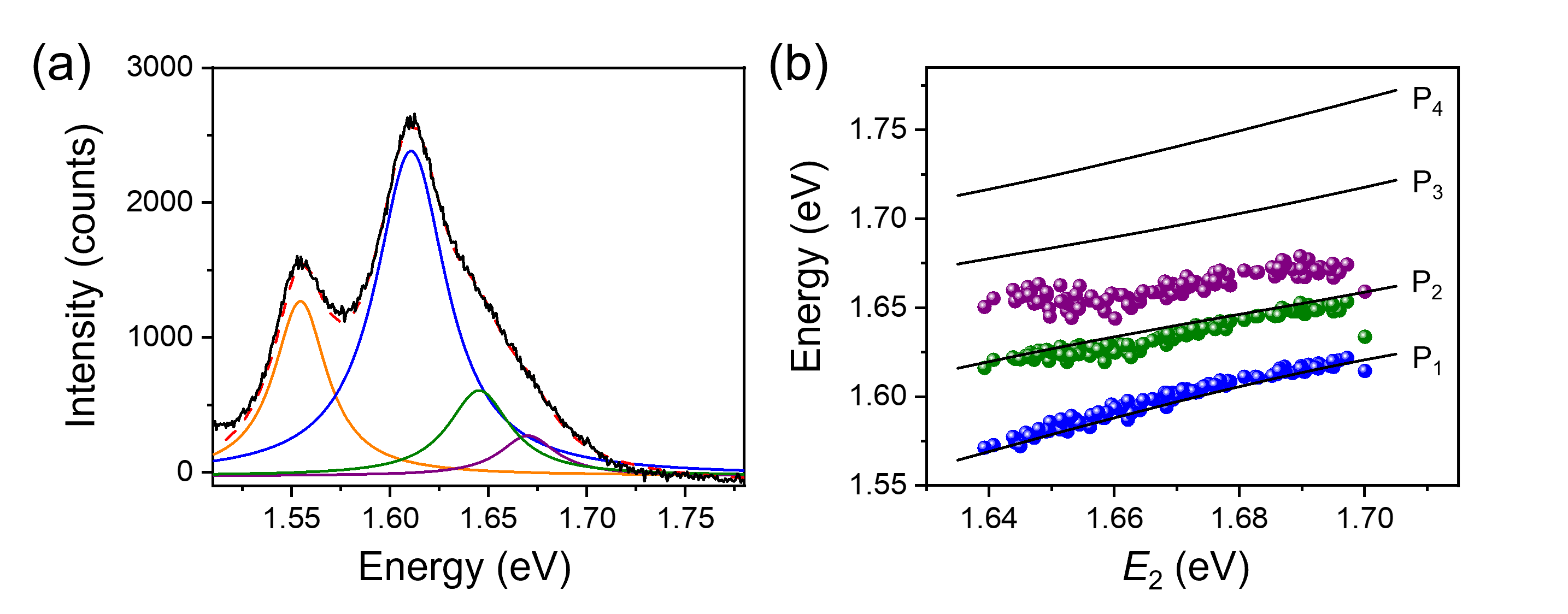


**Figure S7.** ﻿(a) PL spectrum of a coupled system and its fit by four Lorentzian peaks. *E*_2_ = 1.683 eV. (b) Energies of fitting peaks (blue, green, and purple) in the experimental PL spectra as a function of *E*_2_. The black lines are the calculated results in Figure 2b.


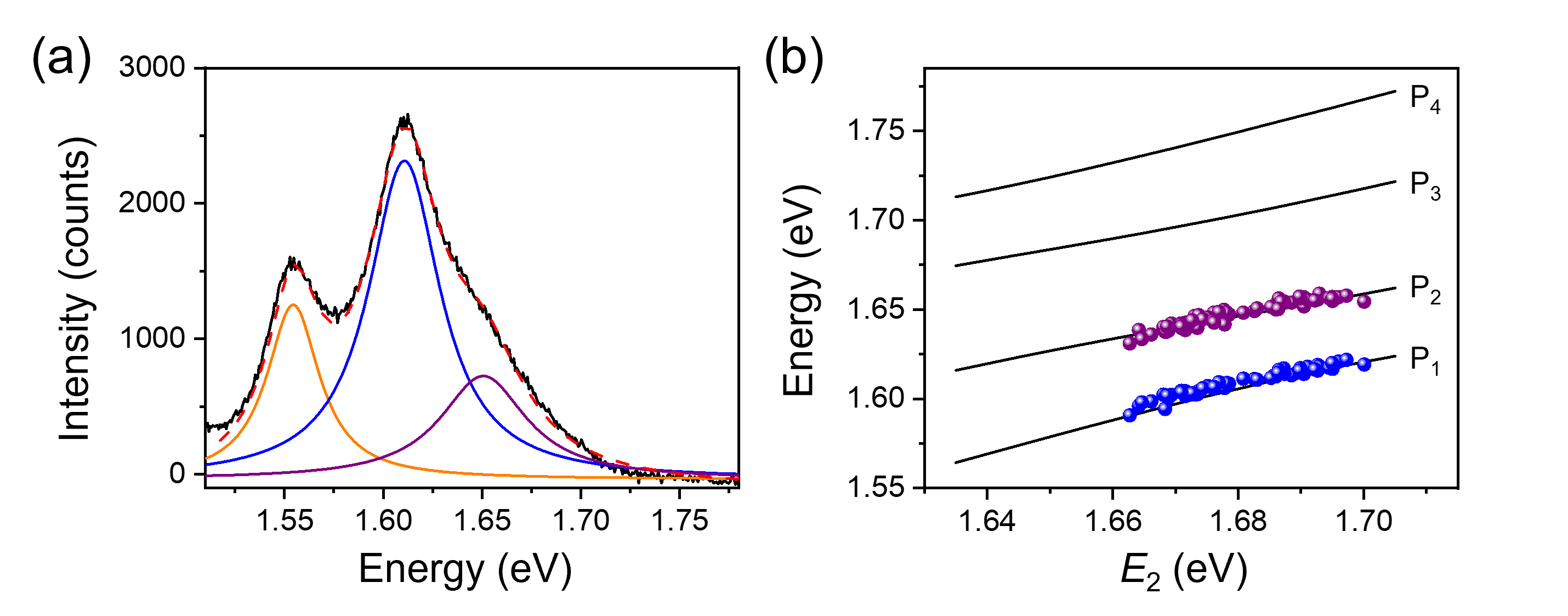


**Figure S8.** ﻿﻿(a) PL spectrum of a coupled system and its fit by three Lorentzian peaks. *E*_2_ = 1.683 eV. (b) Energies of fitting peaks (blue and purple) in the experimental PL spectra as a function of *E*_2_. The black lines are the calculated results in Figure 2b.


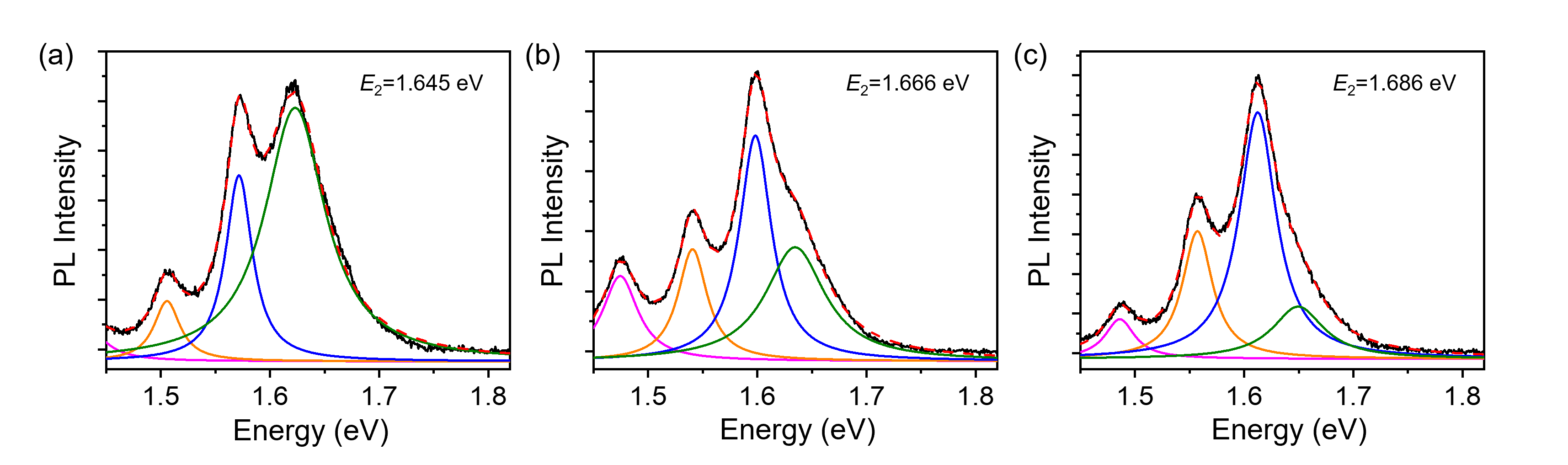


**Figure S9.** Three PL spectra from Figure 3a fitted by multiple Lorentzian peaks.

For the PL spectra of the coupled systems, we firstly assume that they are composed of peaks corresponding to SP_L_, P_1_, and P_2_, and uncoupled excitons and fit them by four Lorentzian peaks (Figure S7a). The energies of the three higher-energy fitting peaks are plotted as a function of the SP_2_ energy *E*_2_, as shown in Figure S7b. The blue and green dots are distributed close to the black lines of P_1_ and P_2_ for scattering, respectively, but the purple dots don’t keep a constant energy and have a similar dependence on *E*_2_ as the green dots. This indicates that the PL intensity of uncoupled excitons is much lower compared with that corresponding to P_2_, and the peak of true P_2_ is divided into the green and purple peaks in the fitting. For the spectra of the coupled systems with large *E*_2_, P_2_ is vanished in the scattering spectra, as can be seen from the bottom spectrum in Figure 2a. We assume that the PL spectra for these coupled systems are composed of SP_L_, P_1_, and uncoupled excitons and fit them by three Lorentzian peaks (Figure S8a). Figure S8b shows the energies of the two higher-energy fitting peaks as a function of the SP_2_ energy *E*_2_. The blue and purple dots are distributed close to the black lines of P_1_ and P_2_ for scattering, respectively, which indicates that the purple fitting peak corresponds to P_2_ rather than uncoupled excitons. When all of the PL spectra are fitted by three Lorentzian peaks, the energies of the two higher-energy peaks agree with the P_1_ and P_2_ branches obtained from the scattering spectra and the lowest energy peak agrees with the SP_L_ peak in the corresponding scattering spectra, as shown in Figure 3b. These results indicate that the weight of uncoupled excitons in the detected PL signal is very low. Figure S9 shows three PL spectra fitted by multiple Lorentzian peaks where the green, blue, and orange peaks correspond to P_2_, P_1_, and SP_L_, respectively.

1. Results for different excitation powers


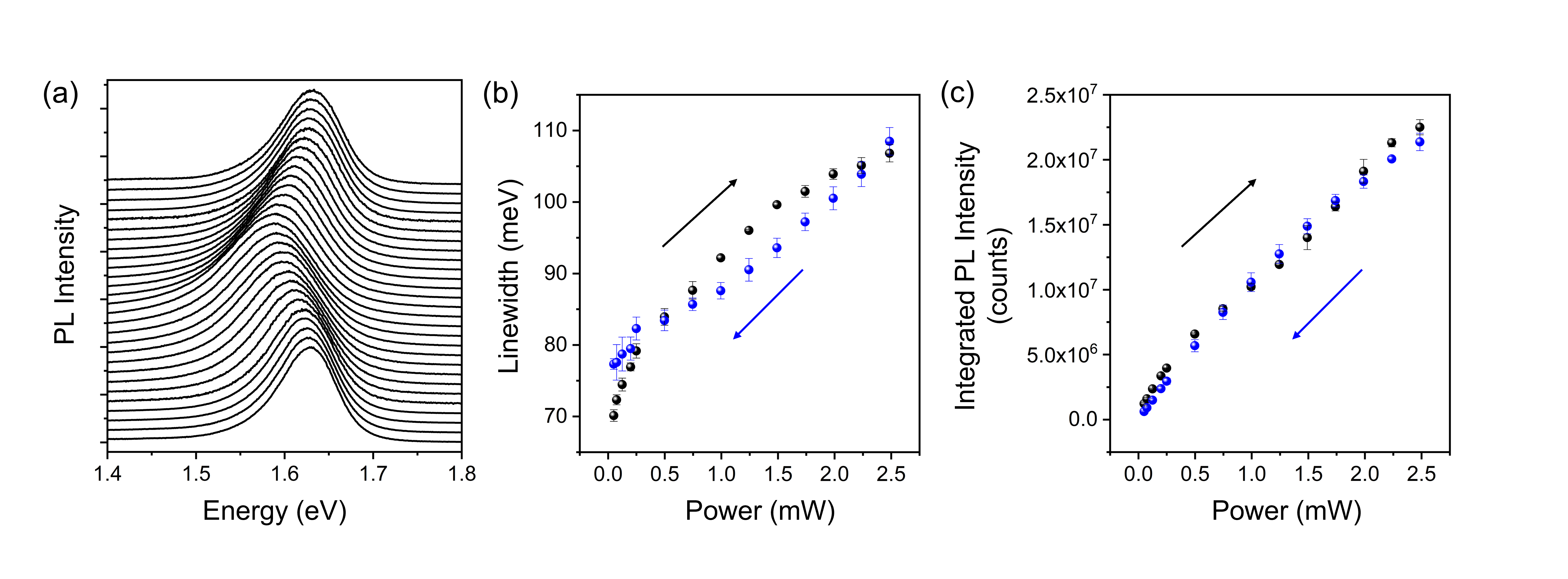


**Figure S10.** (a) Power dependent PL spectra of bare monolayer WSe_2_. The excitation power is increased from 0.05 mW to 2.49 mW and then decreased to 0.05 mW gradually from bottom to top. The thickness of Al_2_O_3_ is 15 nm. The PL spectra are normalized by the maximum intensity and offset for clarity. (b) Linewidth of the PL spectra of bare WSe_2_ as a function of excitation power corresponding to (a). (c) Integrated intensity of the PL spectra of bare WSe_2_ as a function of excitation power. The integration time is 0.5 s. The black and blue arrows show the processes of increasing and decreasing the excitation power, respectively. The black and blue dots are the average values obtained from the spectra measured at three positions on the same WSe_2_ monolayer, and the error bars represent the standard deviation.


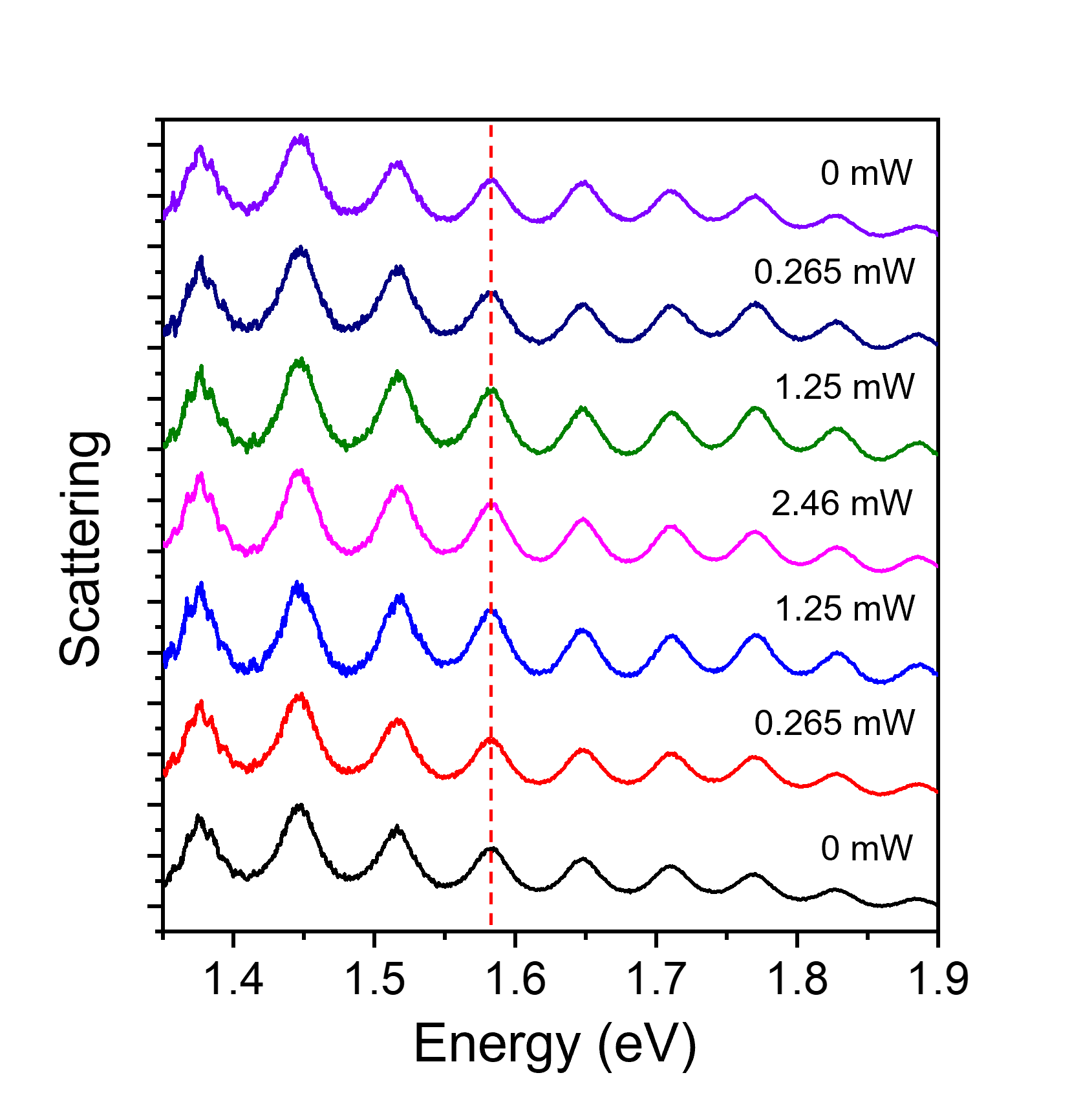


**Figure S11.** Scattering spectra of a Ag NW on glass substrate under the illumination of 532 nm laser light with different powers. The power of 532 nm laser light is increased from 0 to 2.46 mW and then decreased to 0 gradually from bottom to top. The polarization of 532 nm laser light is parallel to the NW. The thickness of Al_2_O_3_ is 10.6 nm. The red dashed line marks the energy of one SP mode, which shows that the SP resonance energies are not affected by the 532 nm laser light.


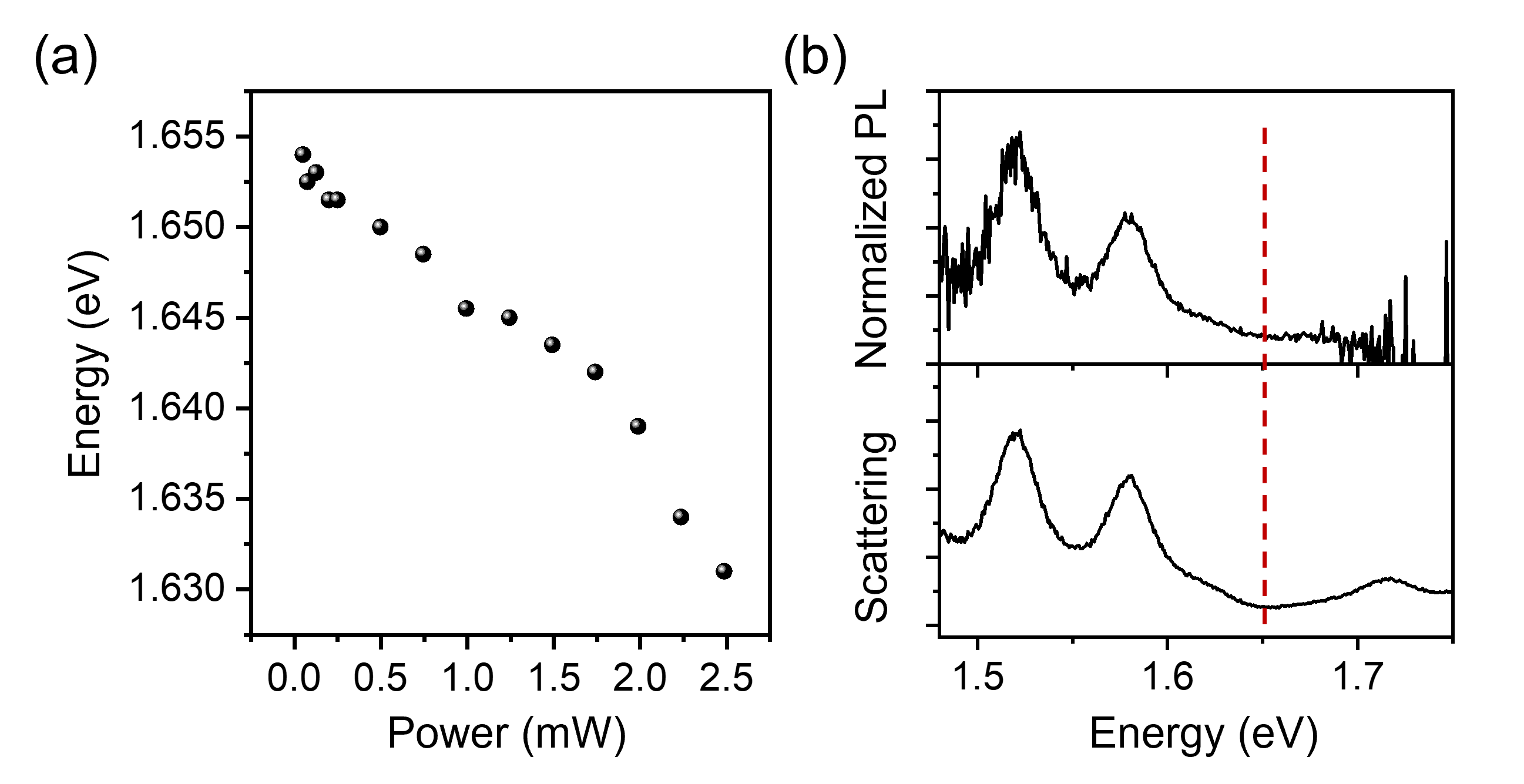


**Figure S12.** (a) Dip energy extracted from Figure 4c as a function of excitation power. (b) Normalized PL spectrum (for excitation power of 0.05 mW) and the corresponding scattering spectrum. The dips are marked with the red dashed line.

The dip in the scattering spectra of the strong coupling system reflects the transmission dip of monolayer WSe_2_. Since the normalized PL spectra show the same profiles as the corresponding scattering spectra, we can extract the dip energy values from the normalized PL spectra for different excitation powers as the transmission dip energy of the monolayer WSe_2_. As shown in Figure S12a, the dip is redshifted with the increase of the excitation power. At the low excitation power, the dips in the normalized PL spectrum and the corresponding scattering spectrum are at the same energy, as shown in Figure S12b (see also Figure 3c).

**References**

[1] Y. Sun and Y. Xia, "Large-scale synthesis of uniform silver nanowires through a soft, self-seeding, polyol process", *Adv. Mater.*, vol. 14, no. 11, pp. 833-837, 2002.

[2] H. Li, G. Lu, Y. Wang*, et al.*, "Mechanical exfoliation and characterization of single- and few-layer nanosheets of WSe_2_, TaS_2_, and TaSe_2_", *Small*, vol. 9, no. 11, pp. 1974-1981, 2013.
